# Enhanced biomass and desulfurization by recombinant *Rhodococcus qingshengii* IGTS8 in bioreactor cultures

**DOI:** 10.1007/s00253-026-13804-2

**Published:** 2026-04-06

**Authors:** Olga Martzoukou, Dimitra Breyanni, Alexander L. Savvides, Amalia D. Karagouni, Diomi Mamma, Dimitris G. Hatzinikolaou

**Affiliations:** 1 Enzyme and Microbial Biotechnology Unit, Department of Biology, National and Kapodistrian University of Athens, Athens, Greece; 2 Biotechnology Laboratory, Sector of Synthesis and Development of Industrial Processes (IV), School of Chemical Engineering, National Technical University of Athens, Athens, Greece

**Keywords:** Biodesulfurization, Genetic engineering, Stirred-tank bioreactor, Dibenzothiophene, 2-Hydroxybiphenyl, Methionine, *ΔmetB*, Whole-cell biocatalyst

## Abstract

**Abstract:**

*Rhodococcus qingshengii* IGTS8 is a model strain well-known for its ability to remove sulfur from dibenzothiophene (DBT) and its derivatives via the 4S pathway, a process that catalyzes the conversion of DBT to 2-hydroxybiphenyl (2-HBP) and sulfite without losses in the energy content of the fuel. The desulfurization ability of the wild-type (wt) strain is limited due to repression of the *dsz* operon in the presence of bioavailable sulfur sources, including methionine and in some cases, sulfates, whereas genetic deletion of the reverse transsulfuration pathway enzymes, Cbs and MetB, alleviates this effect. Herein, we examined the growth and biodesulfurization activity of two genetically engineered *R. qingshengii* IGTS8 strains, Δ*cbs* and Δ*metB*, when grown in a stirred tank bioreactor in the presence of their preferred sulfur sources, sulfate and methionine, respectively. The Δ*cbs* strain demonstrated the highest biomass concentration, but the lowest desulfurization activity, whereas Δ*metB*, when grown in a repressive sulfur source, exhibited desulfurization activity comparable to that of the wt grown under non-repressive conditions. Investigation of the influence of ethanol and methionine supply for Δ*metB* strain, highlighted carbon, and not sulfur concentration, as the critical factor for more efficient growth and desulfurization activity. The highest rates in batch mode were achieved in the presence of 165 mM ethanol and 2 mM methionine. Finally, fed-batch cultures of Δ*metB* improved desulfurization efficiency up to 99.3% compared to batch mode, upon optimization of ethanol feeding rates. Thus, we identify carbon source as an important factor for process upscale, while dynamic control of nutrient supply may contribute to balancing biomass production and enzymatic activity.

**Key points:**

• *Recombinant R. qingshengii IGTS8 cultivated under sulfur-repression in bioreactors.*

• *Carbon supply limits growth and desulfurization activity in batch cultures.*

• *Fed-batch ethanol control boosts longevity, biomass, and desulfurization activity.*

**Supplementary Information:**

The online version contains supplementary material available at 10.1007/s00253-026-13804-2.

## Introduction

Over the past decades, environmental regulations have pushed the currently used hydrodesulfurization technologies to their limits, especially when recalcitrant thiophenic compounds are concerned. Biodesulfurization (BDS) is the microbial process of sulfur acquisition from recalcitrant organosulfur compounds via the 4S pathway-mediated, selective carbon–sulfur bond cleavage (Kilbane 2016, 2006). The biotechnological importance of the process lies in the extraction of sulfur from compounds commonly present in petroleum such as dibenzothiophene (DBT) and its derivatives, without carbon–carbon bond cleavage and thus, without a reduction in the calorific value of the fuel. *R. qingshengii* IGTS8 is the most extensively studied Gram-positive desulfurizing strain to date, that exhibits the biocatalytic properties of interest (Gallagher et al. 1993; Gray et al. 1996; Thompson et al. 2020). Central to the process are four enzymes, namely DszABCD, that catalyze the conversion of DBT to 2-hydroxybiphenyl (2-HBP) and sulfite ions via the four-step 4S pathway (Denome et al. 1993) (Supplementary Information, Fig. S1). Biodesulfurization is subject to several limitations, including the repression of the desulfurization operon *dszABC* under the control of its native promoter *P*_*dsz*_, when the sulfur-containing amino acids methionine and cysteine are supplied in the culture medium (Li et al. 1996; Martzoukou et al. 2022; Tanaka et al. 2002). The most common practice for achieving high catalytic rates is the acquisition of large cell biomass under non-repressive conditions, followed by the utilization of the biocatalyst in the form of resting cells in DBT desulfurization assays (Mohebali and Ball 2016). However, most of the BDS-related research has focused on small-scale biocatalyst growth (in orbital shakers) for consequent applications in the form of resting cells, with a more limited number of studies conducted in bioreactor configurations. The advancement of BDS necessitates the transfer of all knowledge gained from work at the flask scale to the bioreactor scale, whilst other factors should also be considered (El‐Gendy and Nassar 2018).

At the process level, operational and nutritional parameters play a pivotal role in achieving maximum biodesulfurization efficiency, including but not limited to reaction time, concentration and age of the biocatalyst, oil phase fraction, initial sulfur concentration, and medium composition (del Olmo et al. 2005a, b; El‐Gendy and Nassar 2018; Hokmabadi et al. 2022; Martin et al. 2005). In addition, oxygen mass transfer is considered an important factor in biodesulfurization process development that has been studied using airlift and stirred tank bioreactors (Boltes et al. 2013; Garcia-Ochoa and Gomez 2009; Martin et al. 2005; Martinez et al. 2016; Zhang et al. 2013), whereas maintaining a constant oxygen transfer rate (OTR) and volumetric oxygen mass transfer coefficient (kLa) has also been reported as a promising strategy for desulfurization scale-up (Boltes et al. 2013; Garcia-Ochoa and Gomez 2009; Martinez et al. 2016). Several studies have employed the model strain *R. qingshengii* IGTS8 (Abin-Fuentes et al. 2014; del Olmo et al. 2005b; Honda et al. 1998; Santos et al. 2006; Schilling et al. 2002) or other desulfurizing *Rhodococci* (Jatoi et al. 2021; Maass et al. 2014; Wang et al. 1996; Wang and Krawiec 1996) at the bioreactor scale, highlighting the influence of both operational and nutritional parameters in achieving maximum biodesulfurization rates. Furthermore, the use of magnetic nanoparticles was shown to enhance the rate of desulfurization and cell growth in a stirring reactor (Karimi et al. 2017). Gomez et al. (2015) utilized a stirred and sparged tank bioreactor and investigated the dependence of *R. qingshengii* IGTS8 growth and desulfurization capacity on agitation speed, reporting the minimum and maximum speeds for meeting cells oxygen demand without adversely affecting strain performance. Prasoulas et al. (2021) investigated various parameters utilizing *R. qingshengii* IGTS8 in a two-phase bubble column bioreactor, including the age and concentration of cells, the organic phase percentage, as well as the type and concentration of organosulfur compound supplemented. In addition, *R. erythropolis* KA2-5–1 has been employed in exponential fed-batch cultures to investigate the effect of different sulfur sources on the desulfurization activity of the strain (Konishi et al. 2005). An important aspect to consider in process development is the optimal balance between high desulfurization levels and maintaining BDS activity for longer growth times (Mohebali and Ball 2016). Elevated dissolved oxygen levels can result in increased growth rates, though at the expense of BDS capability (Gomez et al. 2006). Conversely, high BDS activity for extended growth periods can be achieved in the presence of lower oxygen saturation levels (del Olmo et al. 2005b).

Genetic engineering approaches could also lead to process enhancement; however, targeted genetic engineering of the model biocatalyst *R. qingshengii* IGTS8 was achieved only recently (Martzoukou et al. 2022, 2023a, 2025). These genome-based modifications are preferable for large-scale applications compared to plasmid-based approaches, as they do not necessitate the supplementation of antibiotics and generate more genetically stable biocatalysts (Liang and Yu 2021). Notably, a recombinant *R. qingshengii* IGTS8 strain harboring a deletion of the cystathionine gamma-lyase gene, *metB*, was documented to mitigate the effect of methionine-based repression and reach desulfurization levels comparable to the wt strain in the presence of the non-repressive sulfur source methionine (Martzoukou et al. 2022). In addition, upon cystathionine beta-synthase depletion (Δ*cbs* strain), *R. qingshengii* IGTS8 was able to desulfurize in the presence of 1 mM sulfate, while the BDS activity of the wt strain was repressed under the same culture conditions. However, there is a lack of data on the testing of recombinant strains of the model biocatalyst *R. qingshengii* IGTS8 at the bioreactor scale, as well as on the use of genetically engineered desulfurizing biocatalysts that harbor genome-based modifications.

In this context, we further explored the growth and desulfurization capability of the Δ*cbs* and Δ*metB* recombinants (Martzoukou et al. 2022) in comparison to the wt strain in batch reactors in the presence of the preferred sulfur source for each strain, with the supplementation of ethanol as the sole carbon source, and at constant stirring speed and air flow. Moreover, aiming at achieving a higher growth rate and higher BDS activity under sulfur-repressive conditions, we investigated the effect of nutritional parameters such as carbon and sulfur source concentrations, by employing the most promising recombinant strain, Δ*metB*, based on its higher overall desulfurization capacity per culture volume. Specifically, we supplemented batch cultures with three concentrations of the preferred carbon source (45, 87, 165 mM ethanol) and three concentrations of the preferred sulfur source (1, 2, 4 mM methionine). Culture biomass was quantified by measuring absorbance at 600 nm (OD_600_) and converting values to dry cell weight (DCW, g/L) using an established calibration curve (Martzoukou et al. 2022). In parallel, strain catalytic activity was monitored through standard resting-cell desulfurization assays. The ethanol concentrations were selected to provide carbon-excess conditions at the highest level (C:S 330:1 at 1 mM sulfur) and stepwise reductions to evaluate the effect of decreasing carbon availability on growth and BDS activity. Furthermore, optimization of the fed-batch culture was investigated by utilizing the same medium composition as in the batch process, while altering the concentration of ethanol. Variations in the medium flow rate resulted in assessment of the effect of ethanol feeding at three rates, presented in the order of investigation (F_1_ – F_3_; 0.89, 1.30, 1.13 g ethanol/h, respectively). The growth and desulfurization activity of the Δ*metB* recombinant strain exhibited a correlation with the concentration of carbon and sulfur source and was also influenced by the growth phase of the batch culture. Importantly, the operational parameters established in this study led to a significant improvement in growth and catalytic properties for the Δ*metB* recombinant strain, when compared to previously reported shaken batch cultures of smaller volumes (Martzoukou et al. 2022).

## Materials and methods

### Strains, chemicals, and inoculum preparation

The three bacterial strains used in this study are listed in Table 1. For inoculum preparation, *R. qingshengii* IGTS8 strains were grown on Luria–Bertani Peptone agar plates [Bactopeptone 1% (w/v), Yeast extract 0.5% (w/v), NaCl 1% (w/v), Agar 1.5% (w/v)] for 72 h, at 30 °C. Cells were collected from 10 plates and placed in a conical centrifuge tube of 50 mL containing sterile sulfur-free Ringer’s solution (pH 7.0). Cells were collected by centrifugation (500 × *g* for 10 min), washed three times and resuspended in 50 mL of the same solution. Biomass was measured by absorbance at OD_600_ using a Multiskan GO Microplate Spectrophotometer (Thermo Fisher Scientific, Waltham, MA, USA). Initial biomass concentration (g/L) was determined based on an established calibration curve, and a value of 0.05 ± 0.005 g/L was applied for all fed-batch cultures.

**Table 1:** Bacterial strains used in this study

Name	Description	Source
*R. qingshengii* IGTS8	DBT-degrading bacterium, wild-type (wt) strain	ATCC 53968
Δ*cbs*	Genetically engineered IGTS8 strain with *cbs* gene deletion	(Martzoukou et al. 2022)
Δ*metB*	Genetically engineered IGTS8 strain with *metB* gene deletion	(Martzoukou et al. 2022)

### Media and culture conditions

A previously developed sulfur-free chemically defined medium (CDM) (Martzoukou et al. 2022) was used throughout all the experiments in the bioreactor for the cultivation of *R. qingshengii* strains. All strains were grown in batch and fed-batch cultures at 30 °C, at constant agitation (450 rpm) and aeration (1 vvm) while the pH was adjusted at 7.0 using 1 M NaOH and 1 M HCl. In fed-batch cultures, ethanol was supplied with CDM containing 2 mM methionine, at feed rates of 19.2, 28.2, and 30.6 mL/h, corresponding to 0.89, 1.30, and 1.13 g ethanol/h, respectively. Ethanol was the sole carbon source in all cases, and its depletion reflected microbial uptake, with negligible evaporation under the controlled bioreactor conditions. The fermenter was a 7 L Labfors 3 Benchtop bioreactor equipped with a condenser at the air-exit (Infors AG, Switzerland), with a culture working volume of 3–6 L, depending on the experiment. Polypropylene glycol 10% (v/v) was automatically added into the bioreactor to eliminate foam generation. Ten mL samples were collected at selected time intervals for biomass and biodesulfurization activity determination.

### Resting-cell preparation, desulfurization assays, HPLC and GC analysis

For resting-cell preparation, 0.15–4 mL of culture broth were centrifuged at 500 × *g* for 10 min. The medium was discarded, and pellets were washed with a S-free Ringer’s buffer (pH 7.0) and resuspended in 0.45 mL of 50 mM HEPES buffer (pH 8.0) to a defined biomass concentration. For desulfurization assays, the above suspensions were split into three equal volume aliquots (0.15 mL) in Eppendorf tubes and processed exactly as described in Martzoukou et al. (2022). Briefly, DBT (0.15 mL, 2 mM) was added to reach a final concentration of 1 mM, and the tubes were incubated at 30 °C and 1200 rpm for 30 min (Thermo Shaker TS-100, BOECO, Germany). Upon reaction termination with the addition of an equal volume acetonitrile (0.3 mL), samples were vortexed vigorously, centrifuged (14000 × *g*; 10 min) and the supernatant was collected for HPLC analysis as previously described (Martzoukou et al. 2023a). An Agilent HPLC 1220 Infinity LC System, equipped with a fluorescence detector and a C18 reversed-phase column, was used for the analysis (Poroshell 120 EC-C18, 4.6 × 150 mm, 4 μm; Agilent). The elution method, at a flow rate of 1.2 mL/min, began with an isocratic phase for 4 min using a 60:40 (v/v) acetonitrile/H_2_O mixture, then transitioned to 100% acetonitrile over a 15-min linear gradient. Fluorescence was measured with excitation at 245 nm and emission at 345 nm. For the quantification of 2-HBP a standard calibration curve was used (linear range, 10 to 1000 ng/mL) (Martzoukou et al. 2022).

Ethanol concentration in filtered (0.45 μm) culture supernatants was determined through GC-FID, in an Agilent 7820 A system using an Agilent DB-BAC2 UI capillary column (30 m, ID 0.32 mm, film thickness 1.2 μm). The method employed was as follows: Sample volume: 1 μL, injector temperature: 220 ^ο^C, split injection at 50:1 split ratio, carrier gas: helium at 2.5 mL/min, detector temperature: 260 °C, oven program: 5 min hold at 40 ^ο^C, linear ramp at 4 ^ο^C/min up to 100 ^ο^C, linear ramp at 20 ^ο^C/min up to 240 ^ο^C, hold for 2 min.

### Growth modeling and statistical analysis

In order to compare *Rhodococcus* growth behavior at various conditions, batch growth was modeled using a basic unstructured logistic kinetic equation:

$$\frac{d{C}_{x}}{dt}= {\mu }_{max}\bullet {C}_{X}\bullet ({C}_{x}^{max}- {C}_{x})$$

where ${C}_{X}$ represents biomass concentration (g/L), ${C}_{x}^{max}$ is the maximum attainable biomass (g/L), and ${\mu }_{max}$ is the apparent maximum specific growth rate [h^−1^ (g/L)^−1^]. The parameters ${C}_{x}^{max}$ and ${\mu }_{max}$ were estimated for each dataset (${C}_{X}$ *versus* time) via non-linear regression using GraphPad Prism 6.1, by fitting into the integrated form of the equation (Martzoukou et al. 2022).

Data are presented as mean ± SEM (standard error of mean). Statistical significance within a single group was assessed using a one-sample t-test. Comparisons between two groups were performed using a two-tailed paired t-test, whereas comparisons among three or more groups were evaluated using one-way ANOVA followed by Tukey’s post hoc test. All analyses were conducted at a 95% confidence level using GraphPad Prism 6.1.

## Results

### Batch cultures of wild-type and recombinant strains

**Fig. 1:**
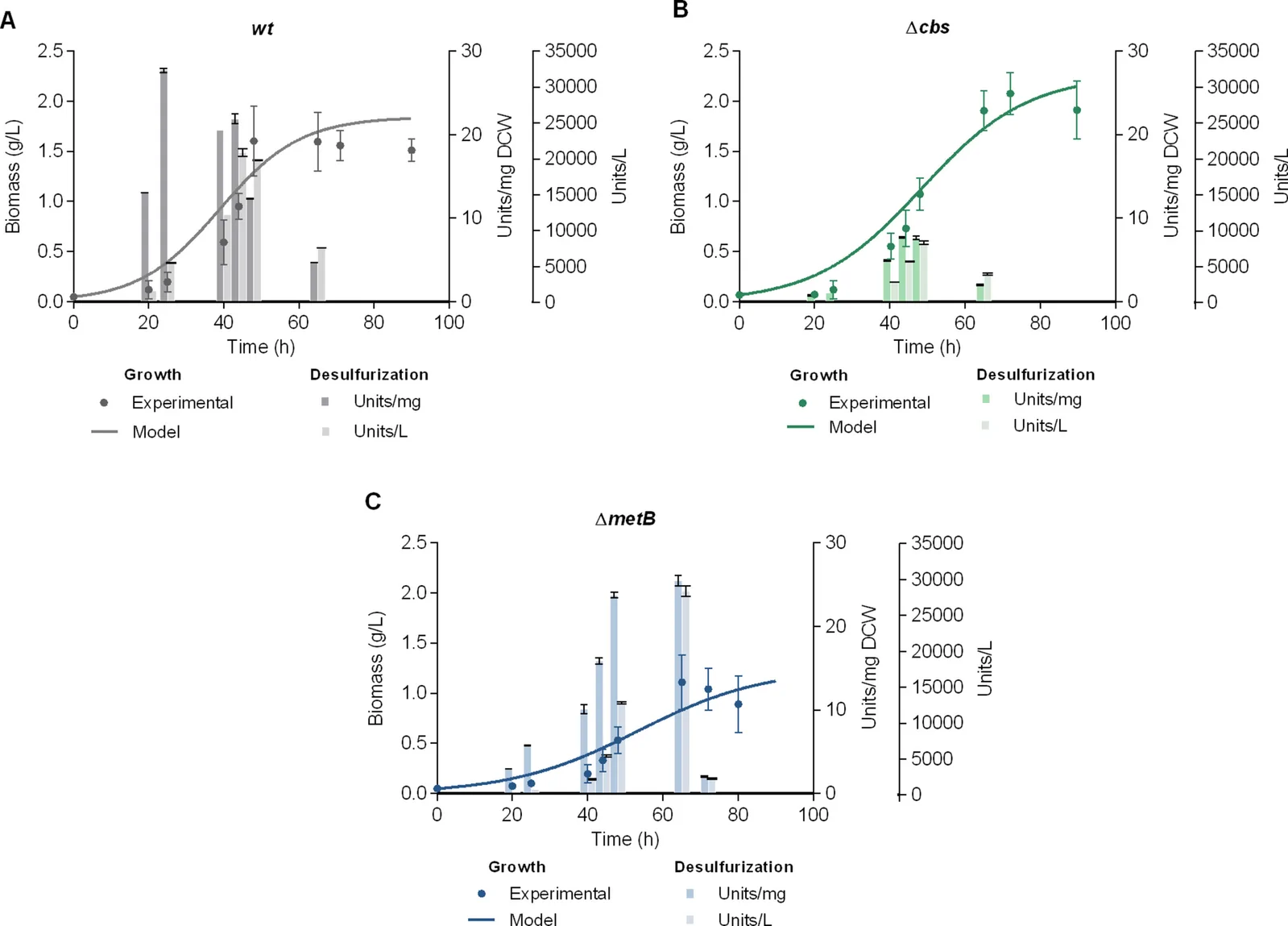
Comparison of the biodesulfurization activities of wt and recombinant strains grown in the presence of their optimum sulfur sources. Growth (Biomass, g/L), specific desulfurization activity (Units 2-HBP/mg Dry Cell Weight [DCW]) and volumetric desulfurization activity (Units 2-HBP/L) of wt **A***,* Δ*cbs* **Β***,* and Δ*metB* **C** strains. All cultures were supplemented with 165 mM ethanol as the sole carbon source. DMSO, sulfate, or methionine (1 mM sulfur) were used as the sole sulfur source for strains wt, Δ*cbs*, and Δ*metB*, respectively. The volume of batch cultures was 5 L in all cases

To investigate the desulfurization potential of Δ*cbs* and Δ*metB* recombinant *R. qingshengii* IGTS8 strains at the bioreactor scale, an initial assessment of growth and resting-cell desulfurization activity was performed in 5 L batch cultures under non-repressive conditions, with the wild-type (wt) strain included for comparison. Each strain was cultivated in the presence of its previously determined optimal sulfur source (1 mM). Dimethyl sulfoxide (DMSO) was used for the wt strain, as methionine, cysteine, and under certain medium compositions, sulfate, are known to repress its desulfurization activity. Sulfate was used for the Δ*cbs* strain, and methionine for the Δ*metB* strain (Fig. 1) (Li et al. 1996; Martzoukou et al. 2022, 2023b). Ethanol (165 mM) was selected as the carbon source for all strains, based on its proven efficacy in promoting growth and desulfurization activity in both wt and recombinant *R. qingshengii* IGTS8, even in the presence of different culture media (Aggarwal et al. 2011; Martzoukou et al., 2022, 2023a, b, 2025). The maximum biomass yield for the wt strain was equal to 1.60 ± 0.35 g_DCW_/L at 48 h of growth, and the maximum specific desulfurization activity was documented at 25 h of growth (27.68 ± 0.24 Units/mg_DCW_, Fig. 1A), albeit in small-scale cultures the respective BDS maximum was documented at the mid-log phase (45 h; 29.03 ± 1.13 Units/mg_DCW_) (Martzoukou et al. 2022). In addition, a reduction of desulfurization activity was observed across the stationary phase. Concurrently, the ethanol consumption curve demonstrated that the carbon source is completely depleted after 48 h, *i.e.*, at the end of the exponential phase in batch cultures, which may account for the reduction of desulfurization activity and the initiation of stationary/death phase (Supplementary Information, Fig. S2). The maximum volumetric activity for the wt strain was documented at the exponential phase, prior to the depletion of ethanol (44 h; 20848 ± 556 Units/L) (Fig. 1A). The recombinant strain Δ*cbs* exhibited the maximum biomass yield (2.08 ± 0.21 g_DCW_/L) at 72 h of growth, the maximum specific desulfurization activity at 44 h (7.71 ± 0.07 Units/mg_DCW_) and the maximum volumetric desulfurization activity at 48 h (8250 ± 251 Units/L) (Fig. 1B). In small-volume cultures of the same strain and under the same medium composition, the specific BDS_max_ was also documented for cells harvested in the mid-log phase and found equal to 15.23 ± 0.27 Units/mg_DCW_ (Martzoukou et al. 2022). Comparison of the two experimental setups in the case of Δ*cbs* underscores a significantly enhanced biomass yield for the bioreactor-scale culture (P < 0.01; Biomass microplate: 1.09 ± 0.03 g_DCW_/L *versus* Biomass bioreactor: 2.08 ± 0.21 g_DCW_/L), which however resulted in a significant decrease of specific desulfurization activity compared to the 96-well plate culture (P < 0.001). The Δ*metB* recombinant strain exhibited maximum biomass yield (1.11 ± 0.27 g_DCW_/L) and maximum specific and volumetric desulfurization activities (25.46 ± 0.65 Units/mg_DCW_ and 28261 ± 716 Units/L, respectively) for cells harvested at 65 h of growth (Fig. 1C), in contrast to the wt and Δ*cbs* strains that reached maximum catalytic activity at earlier growth stages. However, within-strain comparison of the specific desulfurization activities (Units/mg_DCW_) at 48 and 65 h for Δ*metB* indicated only marginal differences, whereas the volumetric desulfurization activity (BDS_vol_, in Units/L; Fig. 1C) appears significantly enhanced at the end of the exponential phase, a fact attributed to the observed increase in biomass concentration (P < 0.0001; Δ*metB*; BDS_vol_ 48 h *versus* 65 h). Small-volume microplate cultures of Δ*metB*, in the presence of 1 mM methionine in the same medium, yielded a BDS_max_ equal to 15.40 ± 0.13 Units/mg_DCW_ (Martzoukou et al. 2022). Batch cultures of Δ*metB* at the bioreactor-scale exhibited significantly enhanced maximum desulfurization activity compared to previously-conducted microplate cultures (P < 0.001) while both maxima were documented at the late-log phase (65 h) (Martzoukou et al. 2022). Notably, comparison between the two different growth setups for each strain revealed an enhancement in specific BDS activity only for the Δ*metB* recombinant strain, whereas the maximum desulfurization activity remained unaffected for the wt strain (P > 0.05), and was significantly reduced for the Δ*cbs* recombinant (P < 0.001), indicating a strain-specific response to the increased culture volume and to the transition from a 96-well plate to a 5 L bioreactor with controlled aeration and pH (Martzoukou et al. 2022 compared to the present study). The recombinant strain Δ*metB*, when grown in the presence of methionine (normally repressive for wt) exhibited maximum specific desulfurization activity comparable to that of the wt strain grown in the presence of the non-repressive sulfur source DMSO (1 mM sulfur; P > 0.05; Δ*metB*, 65 h, 25.46 ± 0.65 Units/mg_DCW_ *versus* wt, 25 h, 27.68 ± 0.24 Units/mg_DCW_). The sulfate-grown Δ*cbs* recombinant strain exhibited the lowest desulfurization activity, reaching a maximum of only 7.71 ± 0.07 Units/mg_DCW_ at 44 h (P < 0.0001; BDS_max_; Δ*cbs versus* wt, Δ*metB*). Comparison of volumetric desulfurization activities highlighted a statistically significant maximum for the Δ*metB* strain at 65 h (1 mM sulfur; P < 0.001 for Δ*metB*, 65 h, 28261 ± 716 Units/L *versus* wt, 44 h, 20848 ± 556 Units/L).

The growth kinetic parameters C_max_ (maximum calculated biomass, g_DCW_/L), and μ_max_ (apparent maximum specific growth rate, h^−1^ (g/L)^−1^) were obtained by fitting the biomass concentration *versus* time experimental values to the logistic equation for all strains and are presented in Table 2.

**Table 2:** Growth kinetic parameters calculated for wt and recombinant *R. qingshengii* IGTS8 strains. All cultures were supplemented with 165 mM ethanol as the sole carbon source. DMSO, sulfate, or methionine (1 mM sulfur) were used as the sole sulfur source for strains wt, Δ*cbs*, and *ΔmetB* respectively. Kinetic parameters were calculated by fitting experimental values for biomass concentration (g/L) versus time (h), to the logistic model. See also Fig. 1. Values represent mean ± SEM

Strain	C_max_ (g_DCW_/L)	μ_max_ [h^−1^ (g/L)^−1^]
wt	1.84 ± 0.53	0.051 ± 0.022
Δ*cbs*	2.21 ± 0.24	0.036 ± 0.007
Δ*metB*	1.25 ± 0.39	0.048 ± 0.023

Notably, Δ*cbs* exhibited the highest C_max_ among the three strains, although the results were non-significant, whereas the biomass yield of Δ*cbs* was significantly higher than that of Δ*metB* (P < 0.01). Contrastingly, Δ*metB* exhibited marginally lower values for the calculated growth kinetic parameters compared to the wt strain (P > 0.05), and a significantly lower biomass yield (P < 0.05), an effect which has been observed previously for small-scale cultures of both recombinant strains (Δ*cbs*, Δ*metB*) in the presence of methionine (Martzoukou et al. 2022).

The comparative analysis of bioreactor performance revealed that while Δ*cbs* achieved higher biomass yields, the superior enzymatic activity of Δ*metB* resulted in greater overall desulfurization capacity per culture volume. Notably, Δ*metB* maintained desulfurization activity levels comparable to the wt strain despite utilizing methionine, an otherwise repressive sulfur source. Medium optimization of large-scale cultures can result in enhanced desulfurization rates, higher biomass yield, and/or prolonged desulfurization activities, and thus the effect of varying methionine and ethanol concentrations was further explored for the Δ*metB* strain

### Effect of methionine concentration on Δ*metB* batch cultures

**Fig. 2:**
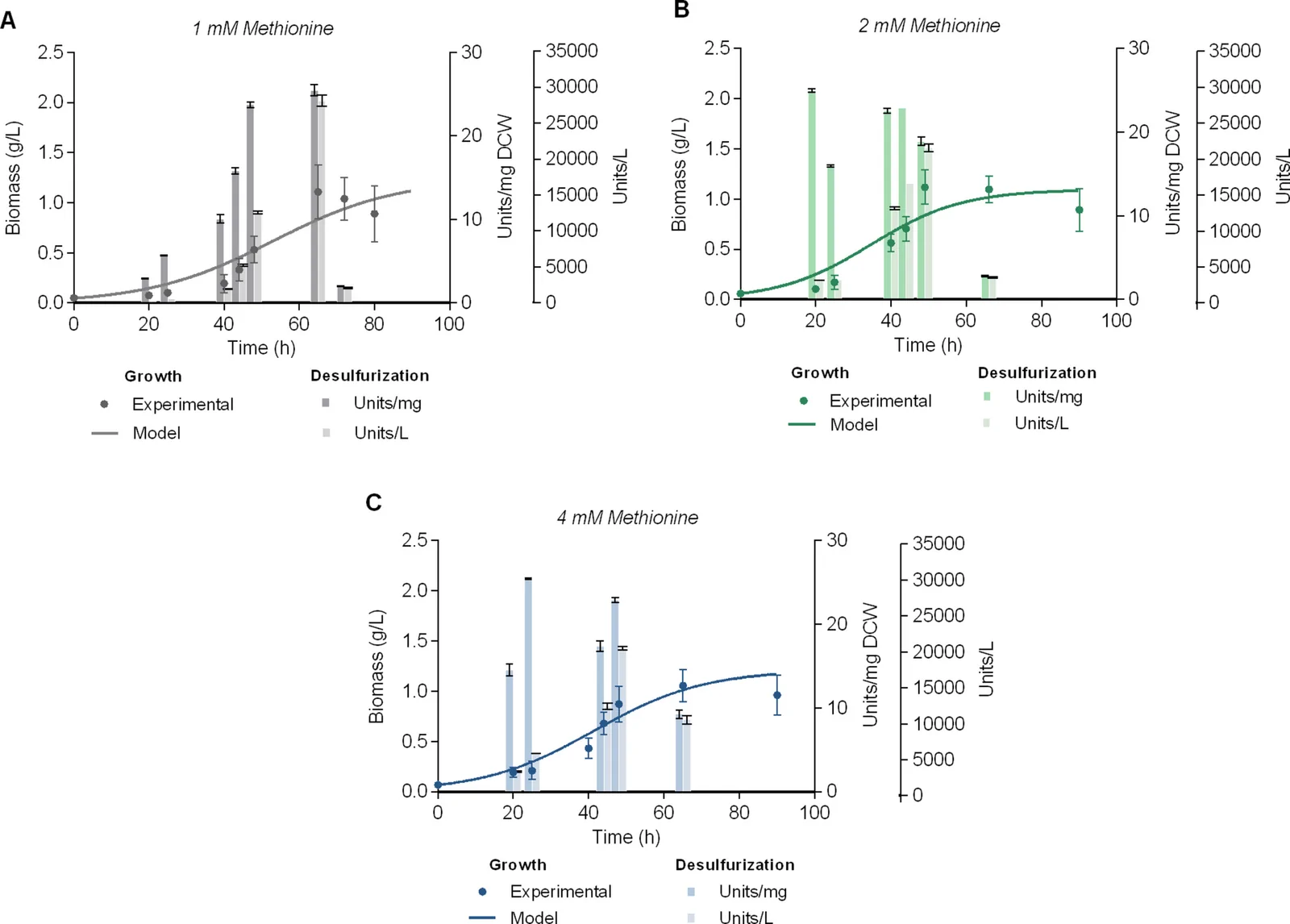
Effect of methionine concentration on Δ*metB* strain growth and resting-cell desulfurization activity. Growth (Biomass, g/L), specific desulfurization activity (Units 2-HBP/mg Dry Cell Weight [DCW]) and volumetric desulfurization activity (Units 2-HBP/L) of Δ*metB* in the presence of 1 mM **A**, 2 mM **Β**, and 4 mM **C** methionine as the sole sulfur source. All cultures were supplemented with 165 mM ethanol as the sole carbon source. The volume of batch cultures was 5 L in all cases. *Please note that *Fig. 1C* is repeated in A*

In the context of bioreactor-scale culture medium optimization studies, we initially investigated the effect of sulfur source concentration on Δ*metB* strain growth and desulfurization activity. Methionine was supplemented at 1, 2, and 4 mM as the sole sulfur source in 5 L bioreactor batch cultures, while 165 mM ethanol was utilized as the sole carbon source in all cases (Fig. 2). Statistical comparison of the obtained biomass yield (1.06–1.12 g_DCW_/L), maximum calculated biomass (C_max_ range: 1.10–1.25 g_DCW_/L, Table 3) and maximum specific desulfurization activity (${\mathrm{BDS}}_{\mathrm{max}}^{\mathrm{Sp}}$ range: 25.00–25.47 Units/mg_DCW_, Table S1) did not reveal any significant differences in the presence of the three sulfur concentrations tested (P > 0.05). However, volumetric biodesulfurization activity was significantly higher in the presence of 1 mM methionine, compared to the other two supplemented sulfur concentrations (P < 0.001). Notably, 2 mM methionine resulted in the highest growth rate (μ_max_ = 0.08 h^−1^ (g/L)^−1^) compared to 1 and 4 mM supplemental methionine (Table 3), and thus, the maximum biomass yield is achieved faster, while the specific desulfurization activity is maintained at high levels throughout the logarithmic growth phase of the culture.

**Table 3:** Kinetic parameters calculated for Δ*metB* strain grown in different culture conditions. Ethanol and methionine were supplemented as the sole carbon and sulfur sources, respectively. See also Fig. 2, Fig. 3

Ethanol (mM)	Methionine (mM)	C_max_ (g_DCW_/L)	μ_max_ [h^−1^ (g/L)^−1^]
45	1	0.68 ± 0.16	0.11 ± 0.04
45	2	0.52 ± 0.10	0.17 ± 0.05
87	2	0.99 ± 0.23	0.09 ± 0.04
165	1	1.25 ± 0.39	0.05 ± 0.02
165	2	1.10 ± 0.15	0.08 ± 0.02
165	4	1.21 ± 0.10	0.06 ± 0.01

### Effect of ethanol concentration on Δ*metB* batch cultures

**Fig. 3:**
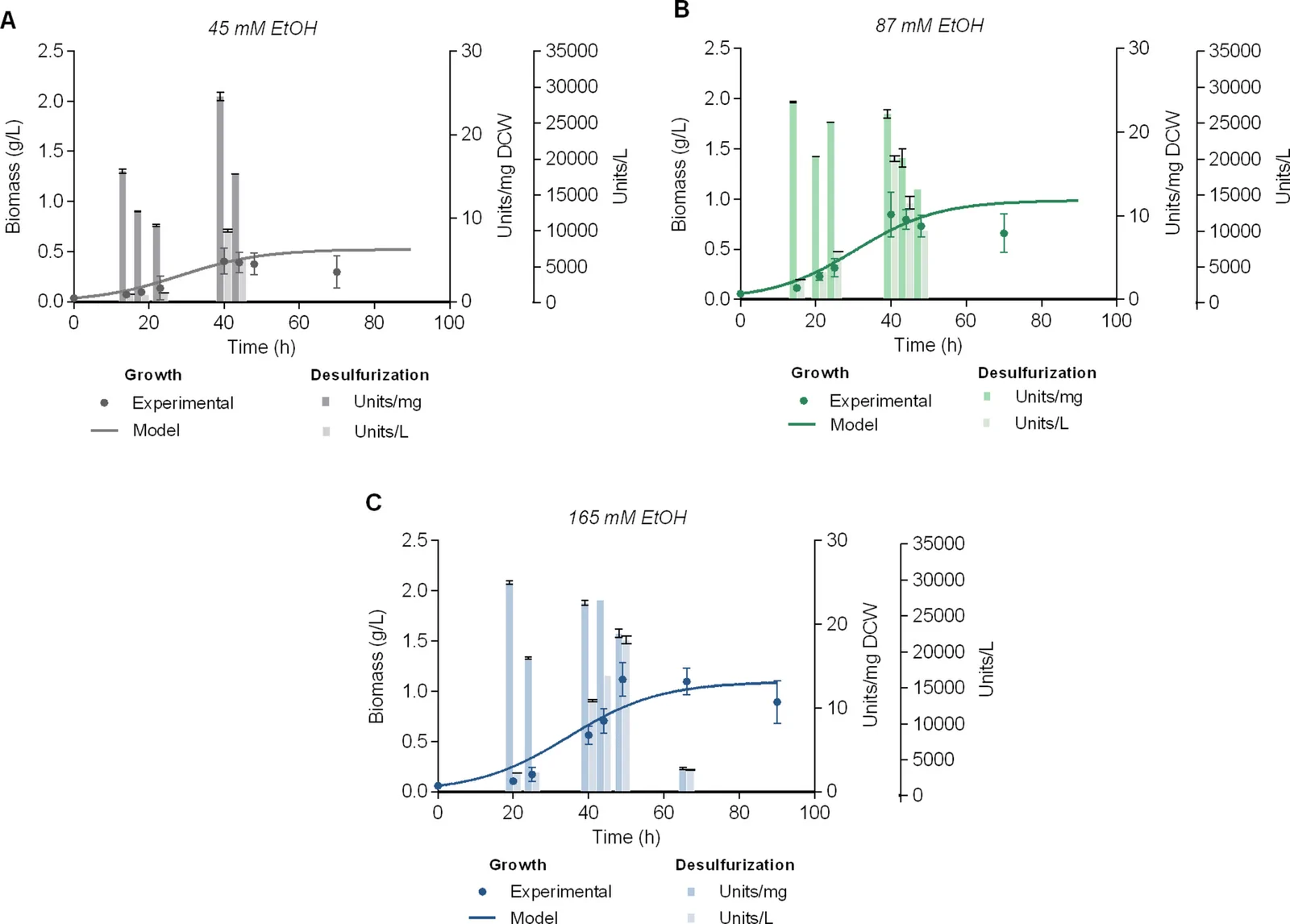
Effect of ethanol concentration on Δ*metB* strain growth and resting-cell desulfurization activity. Growth (Biomass, g/L), specific desulfurization activity (Units 2-HBP/mg Dry Cell Weight [DCW]) and volumetric desulfurization activity (Units 2-HBP/L) of Δ*metB* in the presence of 45 mM **A**, 87 mM **Β**, and 165 mM **C** ethanol as the sole carbon source. All cultures were supplemented with 2 mM methionine as the sole sulfur source. The volume of batch cultures was 5 L in all cases. *Please note that *Fig. 2B* is repeated in C*

Carbon source concentration is known to exert an effect on *R. qingshengii* IGTS8 growth kinetic parameters, while time-dependent variations in desulfurization efficiency have also been reported (Martzoukou et al. 2023a). The effect of ethanol concentration (45, 87, and 165 mM) on Δ*metB* recombinant strain performance was examined using 2 mM methionine as the optimal sulfur source (Fig. 3). Maximum specific desulfurization activity (${\mathrm{BDS}}_{\mathrm{max}}^{\mathrm{Sp}} )$ was not significantly affected by the carbon content (Table S1), albeit at 45 mM ethanol a slightly reduced average desulfurization efficiency was observed (P > 0.05) (Fig. 3 and Table 3). Maximum biomass yield significantly decreased with lower ethanol concentrations (P < 0.01, for 45 mM *versus* 87 mM EtOH; P < 0.01, for 87 mM *versus* 165 mM EtOH; P < 0.0001, for 45 mM *versus* 165 mM EtOH). Furthermore, a correlation was observed between the carbon content and culture duration. For instance, at an ethanol concentration of 165 mM and 2 mM methionine, the maximum biomass yield was attained after 49 h of growth, compared to 40 h for lower concentrations. Volumetric activity (Units/L) was also significantly reduced at 45 mM ethanol (P < 0.0001 *versus* 87 and 165 mM EtOH).

The effect of ethanol concentration was also investigated in the presence of 1 mM methionine. Under these conditions, biomass yield was reduced 2.6-fold, which can be attributed to the lower carbon availability in the medium caused by the ethanol concentration used (P < 0.001, 45 mM *versus* 165 mM EtOH) (Fig. S3 and Table 3). Of note, the culture supplemented with the lower ethanol and methionine concentrations, was also characterized by reduced logarithmic phase duration (40 h, 45 mM EtOH and 1 mM methionine). Table 3 and Table S1 summarize the calculated kinetic parameters and the maximum biodesulfurization activities, respectively, for the Δ*metB* strain grown in the presence of different ethanol and methionine concentrations. Biomass concentrations and culture duration increased with the supplementation of higher ethanol concentrations, whereas the increase in methionine concentration (2 mM) only marginally affected growth rate (μ_max_). Volumetric desulfurization activity exhibited a significant increase in correlation with the carbon concentration. Overall, optimal culture conditions were established at 165 mM ethanol and 2 mM methionine, which balanced biomass production with enzymatic activity, while lower carbon concentrations led to reduced biomass.

### Investigating the effect of ethanol feed rate

A typical problem often observed in batch cultures is the reduction of specific desulfurization activity as the cells exit the logarithmic growth phase, a phenomenon that coincides (but not necessarily correlates) with the depletion of carbon source (Fig. S2). In this context, we assessed the effect of varying ethanol (carbon source) feed rates in fed-batch cultures, in order to investigate the possibility of optimizing Δ*metB* growth characteristics and improving the stability of their desulfurization activity. We used CDM culture medium containing 2 mM methionine as the sole sulfur source. Experiments were initiated as batch cultures at an initial volume V_0_ = 3L and initial ethanol concentration of 165 mM (7.6 g/L). Ethanol feed (in CDM medium containing 2 mM methionine) was applied after 46 ± 2 h, a time point coinciding with the logarithmic phase and a point up to which the desulfurization activity is relatively stable, following what was observed in the corresponding batch process. Feeding continued until the total volume of the culture exceeded 5.5 L. In all cases we monitored culture growth, desulfurization activity, and ethanol levels.

**Fig. 4:**
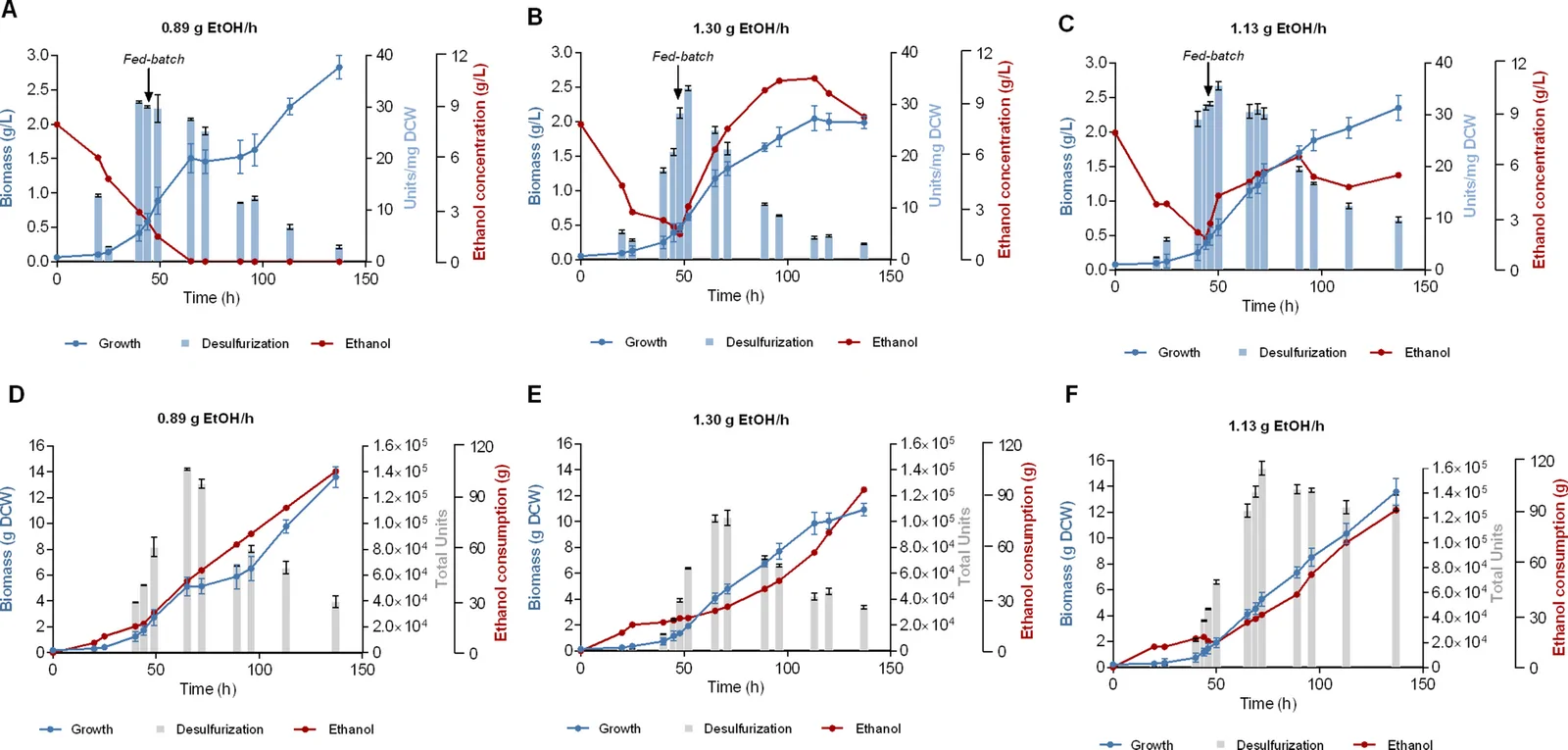
Fed-batch cultures of Δ*metB* strain with varying ethanol feed rates. **A**–**C** Growth (Biomass, g/L), ethanol concentration (g/L), and resting-cell specific desulfurization activity (Units 2-HBP/mg Dry Cell Weight [DCW]) for feed rates of F_1_ = 0.89 *(A),* F_2_ = 1.30 *(Β),* and F_3_ = 1.13 g ethanol/h *(C)*, listed in the order in which they were investigated. **D–F** Growth (Biomass, g_DCW_), ethanol consumption (g), and resting-cell total desulfurization activity (Units 2-HBP/L ∙ culture volume, in L) for the same feed rates as in *(A-C),* respectively. All culture media were supplemented with 2 mM methionine as the sole sulfur source. The volume of initial batch cultures was 3 L and the initial concentration of ethanol was 165 mM (7.6 g/L). The fed-batch processes were initiated at 45, 48, and 46 h, respectively. See also Table S2

Initially we applied a medium feed (F_F_) of 19.2 mL/h at an ethanol (carbon source) concentration of 1 M (46.1 g/L) that corresponds to an ethanol feed of 0.89 g/h (Fig. 4A and D). Under this feeding regime, ethanol concentration in the culture supernatant became undetectable after 60 h (approx. 15 h post-feeding initiation). In an effort to maintain a detectable ethanol concentration in the culture, we tried a second fed-batch experiment keeping all parameters identical but increasing F_F_ to 28.2 mL/h (1.30 g ethanol/h). Notably, this time (Fig. 4B and E) cell growth was unable to consume the added ethanol which started to increase gradually exceeding 10 g/L at the final stages of the experiment. In an effort to optimally control ethanol levels in the fed-batch culture, a third experiment was performed where we aimed for an intermediate value of ethanol feed rate. In this case, we slightly increased the F_F_ to 30.6 mL/h while reducing ethanol feed concentration to 0.8 M (36.8 g/L), thus achieving an ethanol feed of 1.13 g/h (Fig. 4C and F). At this feeding regime, it was possible to achieve some kind of dynamic equilibrium between ethanol feed rate and consumption, that maintained the ethanol concentration in the bioreactor at levels around 5 g/L. Analysis of the three fed-batch cultures indicated a significant improvement in the biodesulfurization activity of the cells for the third feeding regime. Specific BDS activity was notably elevated during the feeding phase, which contributed to sustained high overall activity in the bioreactor throughout the duration of the experiment (P < 0.01, ${\mathrm{BDS}}_{\mathrm{max}}^{\mathrm{Sp}}$ F_3_ *versus* F_1_) (Fig. 4A – C and Table S2). In F_1_ and F_2_, specific BDS reached the maximum at ~ 50 h of growth and gradually declined thereafter. Contrastingly, in F_3_ high BDS values were documented for up to 72 h, in addition to achieving the highest overall values for volumetric and total desulfurization activities (P < 0.01 for ${\mathrm{BDS}}_{\mathrm{max}}^{\mathrm{Vol}}$ and ${\mathrm{BDS}}_{\mathrm{max}}^{\mathrm{Total}}$, F_3_ *versus* F_2_) (Fig. 4D – F and Table S2). Statistical analysis of the experimentally measured biomass concentration highlights a significantly enhanced yield for the feed rate of 0.89 g/h, compared to 1.13 and 1.30 g/h (P < 0.01). The highest biomass value (in grams) was achieved for the F_1_ and F_3_ fed-batch cultures (13.5–13.6 g_DCW_; P < 0.0001 compared to F_2_). Importantly, the intermediate ethanol feed rate (F_3_) resulted in the highest units of desulfurization activity, whereas F_1_ and F_3_ both resulted in the highest biomass yields.

**Fig. 5:**
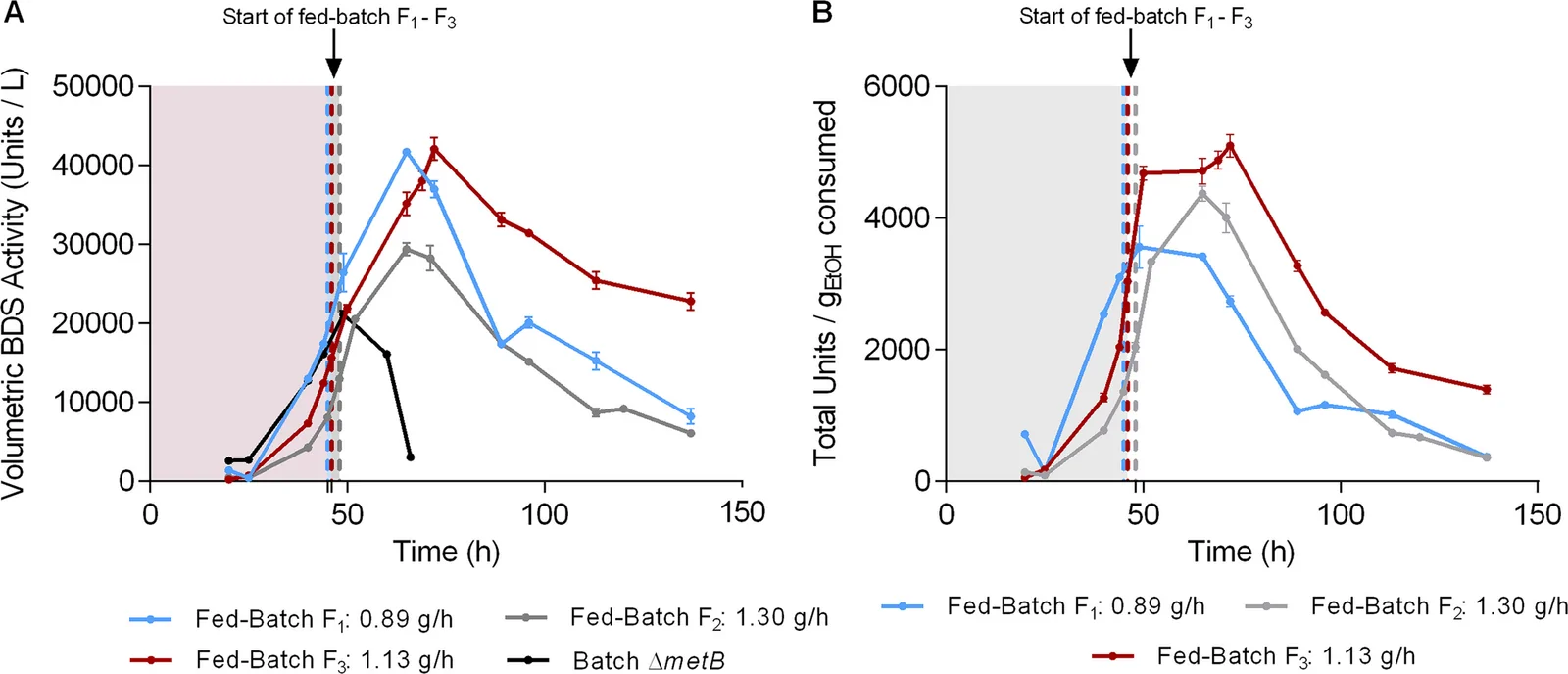
**A** Volumetric desulfurization activity (Units/L) as a function of time (h), and **B** Total desulfurization activity per grams of ethanol consumed, for fed-batch cultures of Δ*metB* strain. The carbon source feeds were 0.89, 1.30, and 1.13 g ethanol/h, and the flow rates were 19.2, 28.2, and 30.6 mL/h for F_1_ – F_3_, respectively. Methionine (2 mM) was the sole sulfur source. The volume of initial batch cultures was 3 L and the initial concentration of ethanol was 165 mM (7.6 g/L). The fed-batch processes were initiated at 45, 48, and 46 h, respectively. The switch to fed-batch is indicated with vertical grided lines. The batch shown in *(A)* was supplemented with 2 mM methionine and 165 mM ethanol

Of more industrial relevance are the comparison data that appear in Fig. 5. Maximum volumetric BDS activities for Δ*metB* strain were markedly increased in all fed-batch cultures when compared to the batch culture (Fig. 5A). For fed batches F_1_ and F_3_, values doubled from 21137 Units/L at 49 h in the batch culture to 42117 Units/L at 72 h in fed-batch F_3_. This corresponds to an almost 40% increase in the total BDS productivity (Units/L/h) at the time-point of maximum volumetric activity, and way higher at later time points. The cellular physiological variations among the different ethanol feeding rates are also reflected in the total desulfurization activity per grams of ethanol consumed. Feeding regime F_3_, where the ethanol concentration was practically maintained constant, proved superior by maintaining the highest volumetric BDS activities and yields with respect to ethanol consumption, throughout the feeding part of the cultures (Fig. 5B).

Comparison of the fed-batch cultures with the batch culture of the same initial carbon and sulfur concentrations, *i.e.*, 165 mM ethanol and 2 mM methionine, highlights a significant enhancement in catalytic activity, in terms of maximum specific, volumetric, and total desulfurization activities (${\mathrm{BDS}}_{\mathrm{max}}^{\mathrm{Sp}} :$ P < 0.01, F_1_ *versus* batch culture; P < 0.001, F_2_ and F_3_ *versus* batch; Table S2). Notably, the volumetric desulfurization activity was up to 99.3% higher for the fed-batch cultures (${\mathrm{BDS}}_{\mathrm{max}}^{\mathrm{Vol}}$: P < 0.001 for F_1_ and F_3_ *versus* batch; P < 0.01 for F_2_ *versus* batch; Table S2), and the total desulfurization activity was up to 51.3% higher (${\mathrm{BDS}}_{\mathrm{max}}^{\mathrm{Total}} :$ P < 0.05 for F_1_ and F_3_ *versus* batch; Table S2).

## Discussion

Despite years of relevant research, biodesulfurization still lacks any short- or medium-term prospects for direct commercial applications. This is primarily because wild-type strains exhibit BDS activities and stabilities that are insufficient for industrial use. Genetic engineering approaches could lead to process enhancement by creating stable and more robust strains (Glekas et al. 2026); however, targeted genetic engineering of the model biocatalyst *R. qingshengii* IGTS8, which had previously been challenging due to its complex genetics, was achieved only recently (Martzoukou et al. 2022, 2023a, 2025). These genome-based modifications are preferred for large-scale applications over plasmid-based methods, as they eliminate the need for antibiotic supplementation and result in biocatalysts with greater genetic stability (Liang and Yu 2021). Building on these advances in genome-based strain engineering, we evaluated the bioreactor-scale performance of two recombinant *R. qingshengii* IGTS8 strains, Δ*metB* and Δ*cbs*, harboring a deletion of either the cystathionine gamma-lyase or cystathionine beta-synthase, respectively (Martzoukou et al. 2022). To our knowledge, Δ*metB* represents the first genome-modified recombinant *R. qingshengii* IGTS8 strain evaluated thoroughly at bioreactor scale, and thus, direct comparisons with previous reactor studies on wt or plasmid-modified strains are limited. Nevertheless, trends observed in volumetric productivity, substrate dependence, and culture longevity are broadly consistent with principles reported in earlier BDS reactor studies using wt strains. In small-scale lab cultures, Δ*metB* was documented to mitigate the effect of methionine-based repression and reach desulfurization levels comparable to the wt strain in the presence of a non-repressive sulfur source, while Δ*cbs* was able to desulfurize in the presence of 1 mM sulfate (a BDS repressing condition for the wt strain).

In bioreactors, the choice of sulfur source critically influenced desulfurization efficiency, fully in line with prior small-volume (0.15 mL) studies (Martzoukou et al. 2022). The non-repressive role of DMSO in the wt strain verifies earlier reports, while sulfate and methionine supported the growth of Δ*cbs* and Δ*metB*, respectively, without enzymatic activity repression, presenting a key advantage for bioreactor scalability. The role of ethanol in sustaining desulfurization was confirmed, and the fact that its depletion correlated with activity decline emphasized the need for further exploring fed-batch strategies. The observed differences in BDS activity maxima between cells of the same strain grown either in small-volume microplate cultures or in bioreactors, can be attributed to differences in operational parameters that affect cell growth and desulfurization efficiency, such as stirring *versus* shaking, and dissolved oxygen availability (Gomez et al. 2006). Specifically, the substantially higher and faster desulfurization activity observed for Δ*metB* in 5 L bioreactor cultures compared to previous microplate experiments (Martzoukou et al. 2022) possibly reflects the combined effects of controlled pH, efficient mixing at larger scale, and improved oxygen transfer. This enhancement was strain-specific, as the wt strain showed similar maximum activity, whereas Δ*cbs* activity was reduced under these conditions. Comparative evaluation between Δ*metB* and Δ*cbs* recombinant strains revealed significant differences in their desulfurization capabilities and growth characteristics, when grown in batch bioreactors in the presence of their optimum sulfur source. While direct comparison with previous reactor-based studies is limited due to the novelty of the recombinant *R. qingshengii* IGTS8 strains, our findings can be contextualized with prior bioreactor BDS reports using other desulfurizing strains (Boltes et al. 2013; Martin et al. 2005; Martínez et al. 2016). These studies emphasize the critical role of operational parameters, such as pH buffering, aeration, stirring, and carbon source supply, in sustaining volumetric desulfurization activity in aqueous resting-cell conditions. Our results extended these observations to recombinant strains, demonstrating that targeted genetic modifications, combined with careful medium and feeding optimization, can achieve enhanced volumetric and specific BDS activity. The Δ*metB* recombinant strain demonstrated superior biodesulfurization potential compared to Δ*cbs*, exhibiting 3.3-fold higher specific activity. The enhanced performance of the Δ*metB* recombinant strain supplemented with methionine, suggests that disruption of the transsulfuration pathway enhances desulfurization under regulated sulfur availability, in contrast to the sulfate-grown Δ*cbs* strain which maintained baseline activity but showed no improvement over small-volume cultures or compared to the wt strain. In addition, the ability of Δ*metB* to achieve wt-equivalent catalytic activity with methionine, which is a repressive sulfur source for the wt, highlighted its genetically engineered advantage. Importantly, the gradual increase in specific desulfurization activity of strain Δ*metB* in a growth-phase dependent manner and the late-log phase activity peak (65 h) underscores the possibility of harvesting high-efficiency whole-cell biocatalysts for extended periods of time, upon optimization of culture conditions. These findings highlight the strain-specific interplay between sulfur metabolism and desulfurization, underlining the need for customized feeding strategies in industrial applications, and establishing Δ*metB* as a promising candidate for further applications due to its combination of efficient desulfurization and ability to utilize an economical sulfur source. The strain's performance suggests successful circumvention of the native sulfur repression mechanisms through metabolic engineering. Notably, the supplementation of different types of sulfur sources (DMSO, sulfate, or methionine) to each strain, in conjunction with the different genetic backgrounds (wt, Δ*cbs*, Δ*metB*) is known to result in sulfur source-driven metabolic adaptations, as well as in different fluxes of the functioning sulfur assimilation pathways, which in turn, can lead to differences in growth kinetics (Hirschler et al. 2021; Martzoukou et al. 2022; Zumsteg et al. 2023). This effect is also noted herein, as was expected, and is thus irrespective of the culture volume.

The consistent specific desulfurization activity of Δ*metB* in the presence of varying methionine concentrations demonstrated the engineered strain’s robust performance under different sulfur availability conditions. While 1 mM methionine yielded optimal volumetric activity, the enhanced growth kinetics observed with 2 mM methionine presented a practical advantage for process efficiency, as faster biomass accumulation can reduce cultivation time without compromising desulfurization capacity. These findings are in line with industrial process requirements where both growth and catalytic activity are critical parameters (Mohebali and Ball 2016). The results suggested that 2 mM sulfur supplementation may offer the best balance between growth kinetics and desulfurization performance for scale-up applications involving the Δ*metB* strain.

The carbon content optimization studies in Δ*metB* batch cultures revealed that ethanol concentration is a critical factor, with 165 mM proving optimal for maintaining stable desulfurization activity and culture longevity. While specific desulfurization activity remained stable for varying carbon concentrations, the reduced biomass production and shorter culture lifespans observed at lower ethanol levels (45 mM, 87 mM) significantly impacted volumetric productivity. These findings confirm that carbon availability is a key limiting factor for process efficiency, as nutrient-limited conditions (45 mM ethanol, 1 mM methionine) maintained high specific desulfurization activity but failed to support sufficient biomass accumulation for optimal volumetric BDS activity output. For sulfur supply, 2 mM methionine provided the best balance between growth rate and desulfurization performance. The minimal impact of methionine concentration on biomass parameters suggests that sulfur availability was not growth-limiting under these conditions, though the slightly elevated growth rate at 2 mM may indicate subtle metabolic benefits. It is worth mentioning that the strong carbon dependence of volumetric productivity underscores the need for careful medium optimization in scale-up applications.

Fed-batch culture studies provided important insights for scale-up considerations. Notably, a static phase appeared in the culture medium in feeding regime F_1_ (low ethanol feeding rate), likely underscoring the adaptation of cells to ethanol depletion. Biomass concentration continued to increase until the end of the experiment consuming all added ethanol, but the specific desulfurization activity of the cells and the corresponding volumetric activity gradually decreased with time, most probably due to the rapid increase in biomass, and/or the carbon source starvation of the cells. In addition, despite the fact that all fed-batch cultures exhibited an increase in biomass, ethanol accumulation was observed for the F_2_ feeding regime (high ethanol feeding rate), indicating that the supplemented carbon source exceeded the culture requirements during the exponential phase. This high ethanol concentration appeared to have an adverse effect on cell growth, by slowing the rate of biomass accumulation compared to other feeding conditions, and also on the volumetric desulfurization activity, which rapidly started to decrease with increasing ethanol concentration. The nonlinear response to feeding rates (with both under- and over-feeding causing > 25% activity reductions) highlights the need for precise stoichiometric control of the carbon source. The optimal 5 g/L ethanol concentration identified here provides a target for scale-up studies, though the narrow window between depletion (< 1 g/L) and inhibition (> 10 g/L) suggests advanced feeding controls may be necessary for industrial implementation. Despite this limitation, it is established that the optimization of carbon source availability represents an effective strategy for acquiring higher levels of biomass, in the form of resting cells, that exhibit significantly increased catalytic activity for prolonged periods of time.

Compared to batch operation in 5 L volumes, fed-batch cultivation of Δ*metB* using 3 L initial volumes achieved higher specific, volumetric, and total desulfurization efficiency and improved the culture longevity as well as biomass yield, possibly highlighting carbon source availability as the determining factor for growth and desulfurization capacity. However, it is also possible that the smaller initial batch volumes resulted in improved mass transfer and aeration efficiency, highlighting the need to also assess the effect of operational parameters, such as stirring speed and dissolved oxygen availability. The 99% increase in volumetric activity coupled with a 2.5-fold biomass increase, achieved through balanced feeding, demonstrated the potential for substantial process intensification, fully in line with previous reports for enhanced volumetric activity and growth in fed-batch cultures of wt *R. qingshengii* IGTS8, compared to the batch mode (Dimos et al. 2025). Notably, the maintenance of > 25 Units/mg_DCW_ specific activity for 72 h, especially compared to the typical 50-h window in batch systems, suggests fed-batch operation may overcome the activity decline observed in the stationary phase of the batch cultures. The data established that precise optimization of the feeding strategy may provide additional benefits for recombinant strain performance, as insufficient ethanol supply limited desulfurization capacity in batch cultures, while an excessive feeding rate caused ethanol accumulation and growth limitation. These findings underscore the complex interplay between nutrient supply, cell density, culture time, and metabolic activity that must be carefully managed in process design. Comparison with previous studies highlights the importance of further optimizing operational parameters, including aeration, stirring speed, and culture volume, to maximize both biomass accumulation and sustained desulfurization activity (Martin et al. 2005; Martinez et al. 2016). Such optimization is particularly critical for recombinant strains, where genetic modifications may alter substrate uptake and metabolic fluxes relative to wt strains.

In addition, from an industrial perspective, biodesulfurization is typically performed using resting cells rather than actively growing cultures. Biomass is produced in aqueous media with polar carbon sources, harvested, and then applied in batch or continuous biphasic systems where the organic phase contains the sulfur compounds. This strategy is more practical and economically feasible than growing cells directly in petroleum fuels or DBT-containing media, which are poorly water-soluble, expensive, and inhibitory due to accumulation of 2-HBP (*R. qingshengii* IGTS8 growth inhibited at > 200 μM 2-HBP; Dsz enzyme activity reduced at 50–110 μM 2-HBP) (Abin-Fuentes et al. 2013; Honda et al. 1998). Resting cells allow high levels of 4S-pathway enzymes to be expressed during growth, ensuring maximal catalytic activity upon contact with the oil phase, representing the most cost-effective strategy for large-scale BDS applications (Boltes et al. 2013; Glekas et al. 2026; Mohebali and Ball 2016).

Future work should focus on dynamically adjusting carbon supply based on real-time culture demands in fed-batch and continuous cultures, as well as optimizing aeration and stirring speed to maximize process performance. Particular focus should be given in maintaining the balance between biomass production and enzymatic activity. Furthermore, additional engineered *R. qingshengii* IGTS8 strains should be evaluated under controlled feeding regimes, as their catalytic potential may surpass the current Δ*metB* variant.

## Supplementary Information

Below is the link to the electronic supplementary material.

Supplementary file1 (PDF 555 KB)

## Data Availability

The data and materials supporting the findings of this study are available within the article and in Supplementary Information.
