# Supplementary material for: Enhanced biomass and desulfurization by recombinant Rhodococcus qingshengii IGTS8 in bioreactor cultures
Source: Appl Microbiol Biotechnol. 2026 Apr 6;110(1):132. doi: 10.1007/s00253-026-13804-2 (PMC13056767; doi:10.1007/s00253-026-13804-2)
Supplement: Supplementary file 1 — Supplementary file1 (PDF 555 KB) [file 253_2026_13804_MOESM1_ESM.pdf]

Enhanced Biomass and Desulfurization by Recombinant *Rhodococcus qingshengii* IGTS8 in Bioreactor Cultures

Olga Martzoukou[1,#], Dimitra Breyanni[1,2,#], Alexander L. Savvides[1], Amalia D. Karagouni[1], Diomi Mamma[2, †, *], and Dimitris G. Hatzinikolaou[1, †, *]

[1]*Enzyme and Microbial Biotechnology Unit, Department of Biology, National and Kapodistrian University of Athens, Athens, Greece*

[2]*Biotechnology Laboratory, Sector of Synthesis and Development of Industrial Processes (IV), School of Chemical Engineering, National Technical University of Athens, Athens, Greece*

[#] *equal contribution*
†equal contribution
* Correspondence: dmamma@chemeng.ntua.gr, dhatzini@biol.uoa.gr

Supplementary Figures

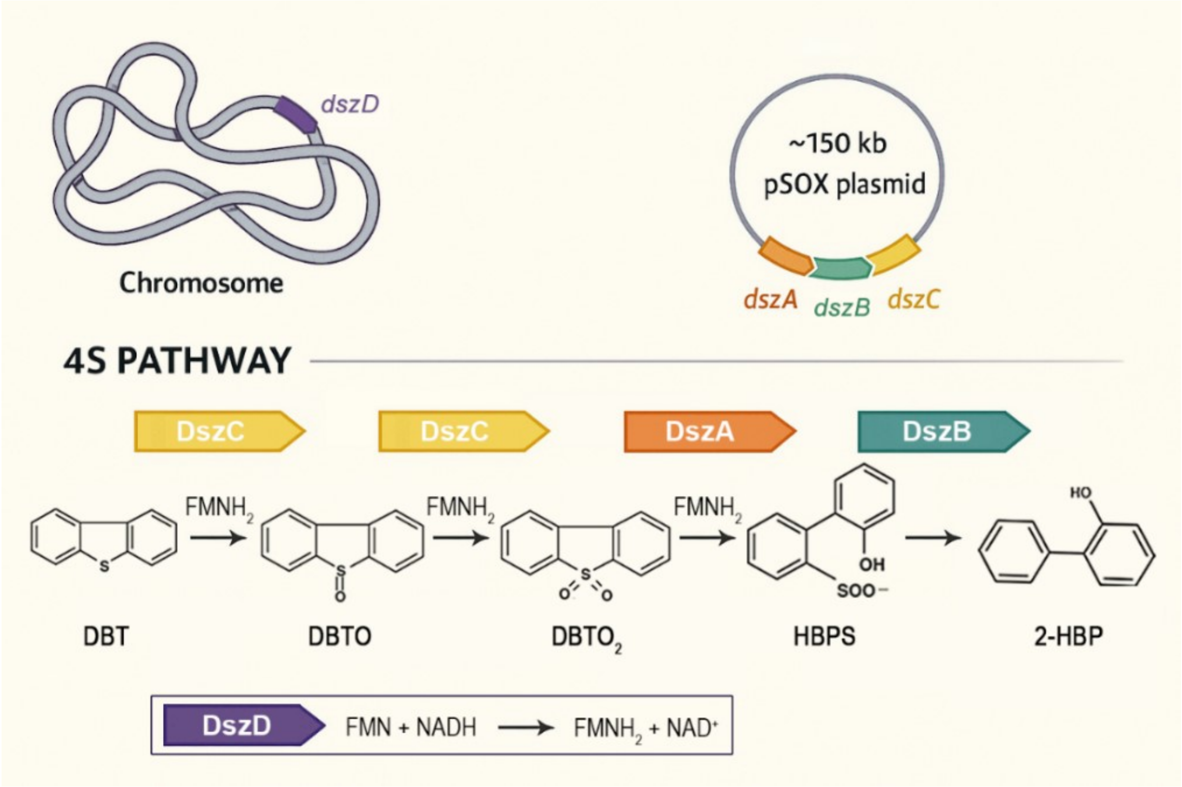

Fig. S1. Genetic and enzymatic background of the 4S desulfurization pathway in *Rhodococcus qingshengii* IGTS8.

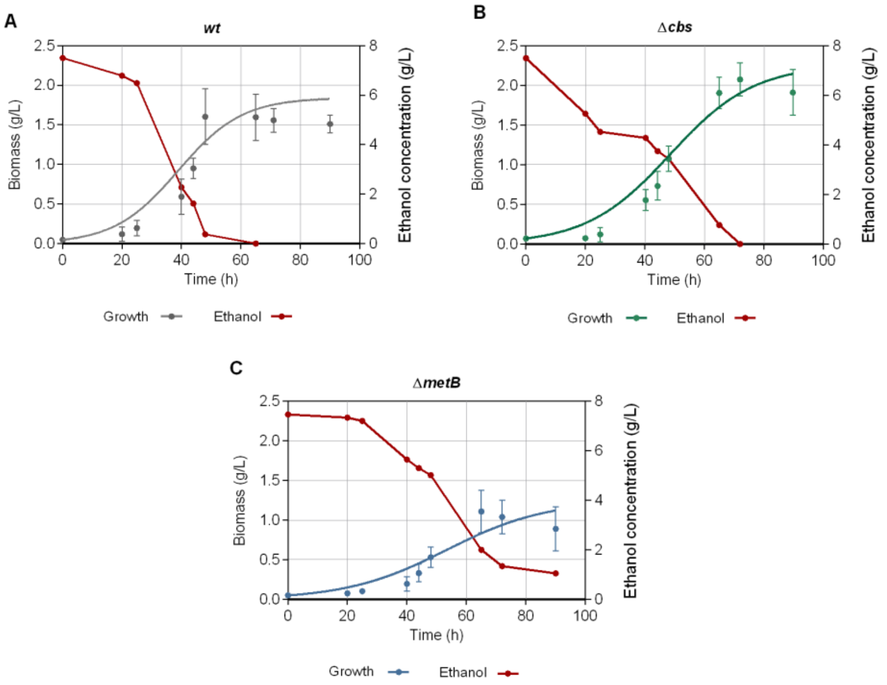

Fig. S2. Growth (Biomass, g/L) and ethanol concentration (g/L) of wt **(A)**, *Δcbs* **(B)**, and *ΔmetB* **(C)** *R. qingshengii* IGTS8 strains cultivated in a 5L batch culture**.** See also Fig. 1.

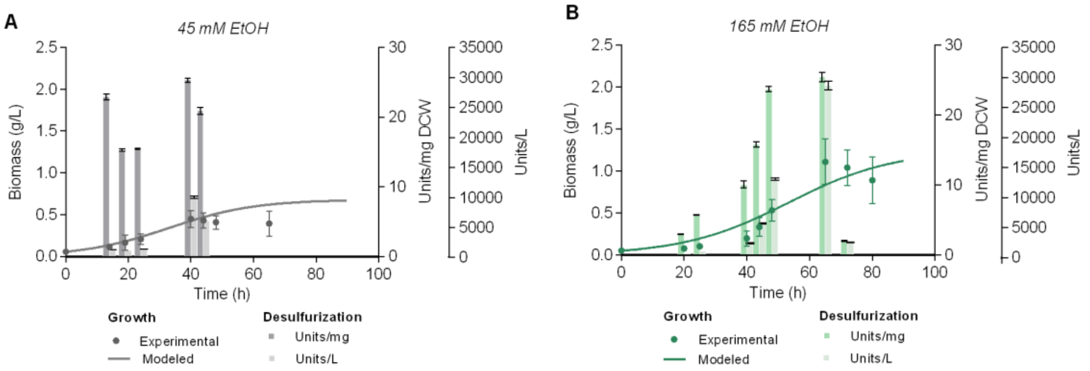

Fig. S3. Effect of ethanol concentration on Δ*metB* strain growth and resting-cell desulfurization activity. **(A – B)** Growth (Biomass, g/L) specific desulfurization activity (Units 2-HBP/mg Dry Cell Weight [DCW]), and volumetric desulfurization activity (Units 2-HBP/L) in the presence of 45 mM *(A),* and 165 mM *(B)* ethanol as the sole carbon source. All cultures were supplemented with 1 mM methionine as the sole sulfur source. The volume of batch cultures was 5 L in all cases.

Supplementary Tables

Table S1. Maximum specific (BDS^{Sp}_{max}) and volumetric (BDS^{Vol}_{max}) biodesulfurization activities, and their respective timepoints (h), for Δ*metB* resting cells grown in the presence of different ethanol (EtOH; carbon source) and methionine (Met; sulfur source) concentrations. See also Figs. 2-3 and Fig. S3.

**EtOH 45 mM, Met 1 mM**		**EtOH 45 mM, Met 2 mM**		**EtOH 87 mM, Met 2 mM**	
BDS^{Sp}_{max} **(Units/mg$_{DCW}$[1])**	BDS^{Vol}_{max} **(Units/L)**	BDS^{Sp}_{max} **(Units/mg$_{DCW}$)**	BDS^{Vol}_{max} **(Units/L)**	BDS^{Sp}_{max} **(Units/mg$_{DCW}$)**	BDS^{Vol}_{max} **(Units/L)**
40 h, 25.33 ± 0.28	40 h, 11399 ± 124	40 h, 24.58 ± 0.53	40 h, 9929 ± 215	15 h, 23.61 ± 0.07	40 h, 19634 ± 427
**EtOH 165 mM, Met 1 mM**		**EtOH 165 mM, Met 2 mM**		**EtOH 165 mM, Met 4 mM**	
BDS^{Sp}_{max} **(Units/mg$_{DCW}$)**	BDS^{Vol}_{max} **(Units/L)**	BDS^{Sp}_{max} **(Units/mg$_{DCW}$)**	BDS^{Vol}_{max} **(Units/L)**	BDS^{Sp}_{max} **(Units/mg$_{DCW}$)**	BDS^{Vol}_{max} **(Units/L)**
65 h, 25.46 ± 0.65	65 h, 28261 ± 716	20 h, 25.00 ± 0.21	49 h, 21137 ± 521	25 h, 25.47 ± 0.08	48 h, 19962 ± 256

[1]DCW, dry cell weight.

Table S2. Maximum specific (BDS_{max}^{Sp}), volumetric (BDS_{max}^{Vol}), and total (BDS_{max}^{Total}) biodesulfurization activities of Δ*metB* resting cells and their respective timepoints (h). Cells were grown in fed-batch cultures supplemented with different ethanol feed rates (g/h). The highest documented values among F1 – F3 are indicated in boldface. See also Figs 4 and 5.

Culture	Ethanol feed (g/h)	BDS_{max}^{Sp} (Units/mg$_{DCW}$)	BDS_{max}^{Vol} (Units/L)	BDS_{max}^{Total} (Units)
**Batch**[1]	-	20 h, 25.00 ± 0.21	49 h, 21137 ± 521	49 h, 105687 ± 2604
**F1**	0.89	40 h, 30.92 ± 0.20	65 h, 41732 ± 266	65 h, 142023 ± 905
**F2**	1.30	52 h, 33.10 ± 0.40	65 h, 29392 ± 776	71 h, 103119 ± 5831
**F3**	1.13	**50 h, 35.59 ± 0.82**	**72 h, 42117 ± 1416**	**72 h, 159860 ± 5376**

[1]Batch culture supplemented with 165 mM ethanol and 2 mM methionine as sole carbon and sulfur sources, respectively. Included for comparison purposes.
